# Rampant software errors may undermine scientific results

**DOI:** 10.12688/f1000research.5930.2

**Published:** 2015-07-29

**Authors:** David A. W. Soergel

**Affiliations:** 1 Department of Computer Science, University of Massachusetts Amherst, Amherst, USA; 2 Current address: Google, Inc., Mountain View, CA, USA

**Keywords:** data management, software error

## Abstract

The opportunities for both subtle and profound errors in software and data management are boundless, yet they remain surprisingly underappreciated. Here I estimate that any reported scientific result could very well be wrong if data have passed through a computer, and that these errors may remain largely undetected.  It is therefore necessary to greatly expand our efforts to validate scientific software and computed results.

## Computational results are particularly prone to misplaced trust

Perhaps because of ingrained cultural beliefs about the infallibility of computation ^1^, people show a level of trust in computed outputs that is completely at odds with the reality that nearly zero provably error-free computer programs have ever been written ^2, 3^.

It has been estimated that the industry average rate of programming errors is “about 15 – 50 errors per 1000 lines of delivered code” ^4^. That estimate describes the work of professional software engineers-—not of the graduate students who write most scientific data analysis programs, usually without the benefit of training in software engineering and testing ^5, 6^. The recent increase in attention to such training is a welcome and essential development ^7– 11^. Nonetheless, even the most careful software engineering practices in industry rarely achieve an error rate better than 1 per 1000 lines. Since software programs commonly have many thousands of lines of code ( Table 1), it follows that many defects remain in delivered code–even after all testing and debugging is complete.

**Table 1.** Number of lines of code in typical classes of computer programs (via informationisbeautiful.net).

Software Type	Lines of Code
Research code supporting a typical bioinformatics study, e.g. one graduate student-year.	O(1000) – O(10,000)
Core scientific software (e.g. Matlab and R, not including add-on libraries).	O(100,000)
Large scientific collaborations (e.g. LHC, Hubble, climate models).	O(1,000,000)
Major software infrastructure (e.g. the Linux kernel, MS Office, etc.).	O(10,000,000)

Software errors and error-prone designs are compounded across levels of design abstraction. Defects occur not only in the top-level program being run but also in compilers, system libraries, and even firmware and hardware–and errors in such underlying components are extremely difficult to detect ^12^.

## How frequently are published results wrong due to software bugs?

Of course, not every error in a program will affect the outcome of a specific analysis. For a simple single-purpose program, it is entirely possible that every line executes on every run. In general, however, the code path taken for a given run of a program executes only a subset of the lines in it, because there may be command-line options that enable or disable certain features, blocks of code that execute conditionally depending on the input data, etc. Furthermore, even if an erroneous line executes, it may not in fact manifest the error (i.e., it may give the correct output for some inputs but not others). Finally: many errors may cause a program to simply crash or to report an obviously implausible result, but we are really only concerned with errors that propagate downstream and are reported.

In combination, then, we can estimate the number of errors that actually affect the result of a single run of a program, as follows:


Number of errors per program execution =
    total lines of code (LOC)
    * proportion executed
    * probability of error per line
    * probability that the error
        meaningfully affects the result
    * probability that an erroneous result
        appears plausible to the scientist.


For these purposes, using a formula to compute a value in Excel counts as a “line of code”, and a spreadsheet as a whole counts as a “program”—so many scientists who may not consider themselves coders may still suffer from bugs ^13^.

All of these values may vary widely depending on the field and the source of the software. Consider the following two scenarios, in which the values are nothing more than educated guesses (informed, at least, by deep experience in software engineering).

### Scenario 1: A typical medium-scale bioinformatics analysis

- 100,000 total LOC (neglecting trusted components such as the Linux kernel).
- 20% executed
- 10 errors per 1000 lines
- 10% chance that a given error meaningfully changes the outcome
- 10% chance that a consequent erroneous result is plausible

Multiplying these, we expect that two errors changed the output of this program run, so **the probability of a wrong output is effectively 100%.** All bets are off regarding scientific conclusions drawn from such an analysis.

### Scenario 2: A small focused analysis, rigorously executed

Let’s imagine a more optimistic scenario, in which we write a simple, short program, and we go to great lengths to test and debug it. In such a case, any output that is produced is in fact more likely to be plausible, because bugs producing implausible outputs are more likely to have been eliminated in testing.

- 1000 total LOC
- 100% executed
- 1 error per 1000 lines
- 10% chance that a given error meaningfully changes the outcome
- 50% chance that a consequent erroneous result is plausible

Here **the probability of a wrong output is 5%.**

The factors going into the above estimates are rank speculation, and the conclusion varies widely depending on the guessed values. Measuring such values rigorously in different contexts would be valuable but also tremendously difficult. Nonetheless it is sobering that some plausible values can produce high total error rates, and that even conservative values suggest that an appreciable proportion of results may be erroneous due to software defects–above and beyond those that are erroneous for more widely appreciated reasons.

Put another way: publishing a computed result amounts to asserting that the likelihood of error is acceptably low, and thus that the various factors contributing to the total error rate are low. In the context of a specific program, the first three factors (# LOC, % executed, and errors/line) can be measured or estimated. However the last two (“meaningful change” and “plausible change”) remain completely unknown in most cases. In the following two sections I argue that these two factors are likely large enough to have a real impact. It is therefore incumbent on scientists to validate computational procedures–just as they already validate laboratory reagents, devices, and procedures–in order to convince readers of the absence of serious bugs.

## Software is exceptionally brittle

A response to concerns about software quality that I have heard frequently—-particularly from wet-lab biologists—-is that errors may occur but have little impact on the outcome. This may be because only a few data points are affected, or because values are altered by a small amount (so the error is “in the noise”). The above estimates account for this by including a term for “meaningful changes to the result”. Nonetheless, in the context of physical experiments, it is tempting to believe that small errors tend to reduce *precision* but have less effect on *accuracy*–i.e. if the concentration of some reagent is a bit off then the results will also be just a bit off, but not completely unrelated to the correct result.

But software is different. We cannot apply our physical intuitions, because software is profoundly brittle: “small” bugs commonly have unbounded error propagation. A sign error, a missing semicolon, an off-by-one error in matching up two columns of data, etc. will render the results complete noise ^16^. It is rare that a software bug would alter a small proportion of the data by a small amount. More likely, it systematically alters every data point, or occurs in some downstream aggregate step with effectively global consequences. **In general, software errors produce outcomes that are *inaccurate*, not merely *imprecise*.**

## Many erroneous results are plausible

Bugs that produce program crashes or completely implausible results are more likely to be discovered during development, before a program becomes “delivered code” (the state of code on which the above errors-per-line estimates are based). Consequently, published scientific code often has the property that nearly every possible output is plausible. When the code is a black box, situations such as these may easily produce outputs that are simply accepted at face value:

- An indexing off-by-one error or other data management mistake associates the wrong pairs of X’s and Y’s ^14, 15^.
- A correlation is found between two variables where in fact none exists, or vice versa.
- A sequence aligner reports the “best” match to a sequence in a genome, but actually provides a lower-scoring match.
- A protein structure produced from x-ray crystallography is wrong, but it still looks like a protein ^16^.
- A classifier reports that only 60% of the data points are classifiable, when in fact 90% of the points should have been classified (and worse, there is a bias in which points were classified, so those 60% are not representative).
- All measured values are multiplied by a constant factor, but remain within a reasonable range.

## Software errors and statistical significance are orthogonal issues

A software error may produce a spurious result that appears significant, or may mask a significant result.

If the error occurs early in an analysis pipeline, then it may be considered a form of measurement error (i.e., if it systematically or randomly alters the values of individual measurements), and so may be taken into account by common statistical methods.

However: typically the computed portion of a study comes after data collection, so its contribution to wrongness may easily be independent of sample size, replication of earlier steps, and other techniques for improving significance. For instance, a software error may occur near the end of the pipeline, e.g. in the computation of a significance value or of other statistics, or in the preparation of summary tables and plots.

The diversity of the types and magnitudes of errors that may occur ^17– 21^ makes it difficult to make a general statement about the effects of such errors on apparent significance. However it seems clear that, a substantial proportion of the time (based on the above scenarios, anywhere from 5% to 100%), a result is simply wrong—-rendering moot any claims about its significance.

## Popular software is not necessarily less bug-prone

The dangers posed by bugs should be obvious to anyone working with niche or custom software, such as one-off scripts written by a graduate student for a specific project. Still it is tempting to think that “standard” software is less subject to these concerns: if everyone in a given scientific field uses a certain package and has done so for years, then surely it must be trustworthy by now, right? Sadly this is not the case.

In the open-source software community this view is known as “Linus’s Law”: “Given enough eyeballs, all bugs are shallow”. The law may in fact hold when there are really many eyeballs reading and testing the code. However widespread *usage* of the code does not produce the same effect. This has been recently demonstrated by the discovery of major security flaws in two extremely widely used open-source programs: the “Shellshock” bug in the bash command line shell and the “Heartbleed” bug in the OpenSSL encryption library. In both cases, code that runs on a substantial fraction of the world’s computers is maintained by a very small number of developers. Despite the code being open-source, “Linus’s Law” did not take effect simply because not enough people read it–even over the course of 25 years, in the case of Shellshock.

This principle applies not only to the software itself, but also to computed results that are reused as static artifacts. For instance, it took 15 years for anyone to notice errors in the ubiquitous BLOSUM62 amino acid substitution matrix used for protein sequence alignment ^22^.

Furthermore, even popular software is updated over time, and is run in different environments that may affect its behavior. Consequently, even if a specific version of a package running on a specific computer is considered reliable, that trust cannot necessarily be extended to other versions of the same software, or to the software when run on a different CPU or on a different operating system ^23^.

## What can be done?

All hope is not lost; we must simply take the opportunity to use technology to bring about a new era of collaborative, reproducible science ^24– 26^. Open availability of all data and source code used to produce scientific results is an incontestable foundation ^27– 31^. A culture of comprehensive code review (both within and between labs) can certainly help reduce the error rate, but is not a panacea. Beyond that, we must redouble our commitment to replicating and reproducing results, and in particular we must insist that a result can be trusted only when it has been observed on multiple occasions using **completely different software packages and methods.**

A flexible and open system for describing and sharing computational workflows ^32^ would allow researchers to more easily examine the provenance of computational results, and to determine whether results are robust to swapping purportedly equivalent implementations of computational steps. A shared workflow system may thereby facilitate distributed verification of individual software components. Projects such as Galaxy ^33^, Kepler ^34^, and Taverna ^35^ have made inroads towards this goal, but much more work is needed to provide widespread access to comprehensive provenance of computational results. Perhaps ironically, a shared workflow system must itself qualify as a “trusted component”–like the Linux kernel–in order to provide a neutral platform for comparisons, and so must be held to the very highest standards of software quality. Crucially, any shared workflow system must be widely used to be effective, and gaining adoption is more a sociological and economic problem than a technical one ^36^. **The first step is for all scientists to recognize the urgent need to verify computational results–a goal which goes hand in hand with open availability of comprehensive provenance via workflow systems, and with verification of the individual components of those workflows.**
