# Supplementary material for: Rampant software errors may undermine scientific results
Source: F1000Res. 2015 Jul 29;3:303. Originally published 2014 Dec 11. [Version 2] doi: 10.12688/f1000research.5930.2 (PMC4629271; doi:10.12688/f1000research.5930.2)
Supplement: Peer review history

# Peer review: Referee response for version 2

**DOI:** 10.5256/f1000research.7365.r9698

**Published:** 2015-10-29

**Authors:** Titus Brown

**Affiliations:** 1 Microbiology and Molecular Genetics, Michigan State University, East Lansing, MI, USA

This is a sober (and sobering) perspective on the likely frequency of software errors, and the author has addressed all of my concerns in the revision.

I have read this submission. I believe that I have an appropriate level of expertise to confirm that it is of an acceptable scientific standard.

# Peer review: Referee response for version 2

**DOI:** 10.5256/f1000research.7365.r9699

**Published:** 2015-08-04

**Authors:** Daniel Katz

**Affiliations:** 1 Computation Institute, University of Chicago, Chicago, IL, USA

This version of the paper is much improved, and in general, I agree with the author's response comments.  I still have some concerns with two issues, and reading the paper made me think of one more point of interest, but in general, I am satisfied to see this paper move forward as accepted.

The first issue I have is in the description of the scenarios, where the first says that in a somewhat typical analysis, the probability of a wrong output is 100%.  This says to me that there is something wrong with the calculations, as I don't think typical outputs are 100% likely to be incorrect.

My second issue is the discussion of workflows in the final paragraph.  While there are indeed a set of computations that are described as workflows, particularly data analysis applications, there is also a large set that are not workflows, particularly simulation applications, that are much better described as monolithic programs (though using subroutines, methods, objects, etc. internally).  I don't think the workflow discussion is needed in this paper, or that it really helps make the key points.

Finally, as an additional point, it would be interesting to compare this paper with work done in fault tolerance (aka resilience) where errors in hardware lead to different types of errors in the application, ranging from hangs and crashes to detectable errors to insignificant or significant undetectable errors.  From my own work, http://www.slideshare.net/danielskatz/aft-dsk-reefinalreviewmay2001 provides an example of this, though there are likely many peer-reviewed published works that could also be used for a source of a comparison.

I have read this submission. I believe that I have an appropriate level of expertise to confirm that it is of an acceptable scientific standard.

# Peer review: Referee response for version 1

**DOI:** 10.5256/f1000research.6338.r7096

**Published:** 2014-12-22

**Authors:** Daniel Katz

**Affiliations:** 1 Computation Institute, University of Chicago, Chicago, IL, USA

This opinion article makes a number of good qualitative points, and while I completely agree that there are errors in most software, I think the chances of those errors leading to incorrect published results are completely unknown, and could potentially be much smaller than the this paper claims.  I think the basic claim in the title and the body of the paper may be dramatically overstated.  The abstract says "most scientific results are probably wrong," but this itself seems wrong.

The author states, "we must insist that a result can be trusted only when it has been observed on multiple occasions using completely different software packages and methods."

First, I think this statement is overly focused on software.  One method for developing trust in results from a particular code is that they match results from other codes.  Another method is that they match results from experiment.  A third method might be based on code review.

Second, this statement is not only true for software, it is also true for this complete paper. In order to believe the chances for errors claimed here, this paper itself needs to be verified, and not at the level of each assumption made internally (in the "How frequently ..." section), but at the level of the overall claim. This is not easy, but it would be worthwhile, similar to the author's statement, "Measuring such values rigorously in different contexts would be valuable but also tremendously difficult" (but at a different level). If "most scientific results are probably wrong," the author should be able to select a relatively small number of papers and demonstrate how software errors led to wrong results. I would like to see such an experiment, which would serve to verify this paper, rather than it standing as an unverified claim about verification.

Finally, there is the classic problem with verification of a model (software, in this case): that fact that it works well in one domain is no guarantee that it will work well in another domain.

Having made these objections to the degree of the illness of the patient, I mostly agree with remedies discussed in the last section.  Open available of data and code is clearly good for both trust and reproducibility.  Running (computational) experiments multiple ways can help finds any errors in any one of them, assuming they do not use common components (e.g., libraries, tools, or even hardware) that could lead to systematic biases.  But how this should be done is less clear.  For example, we have enough workflow systems that I don’t see any need for any one of them to be more trusted than the code that runs on them; we can just use different workflow systems with different code as part of the testing.

Back to the author’s last point, I agree that "to recognize the urgent need" is essential, but to me, the need is verification; I could read this closing comment as saying that the need is widely adopted and widely trusted workflow tools.  This should be clarified.

In summary, this paper could be better titled and less strongly worded in places, and the paper itself needs to be verified.  An alternate title would be one that makes the point, “Software, like other experiments, must be verified to be trusted”

I have read this submission. I believe that I have an appropriate level of expertise to confirm that it is of an acceptable scientific standard, however I have significant reservations, as outlined above.

# Response

**Published:** 2015-07-20

**Authors:** David Soergel

Thanks very much for your insightful comments, and apologies for the long-delayed response.  I believe I have addressed the main point about softening the claims throughout the paper.  Some further thoughts follow:

*"First, I think this statement is overly focused on software.  One method for developing trust in results from a particular code is that they match results from other codes.  Another method is that they match results from experiment.  A third method might be based on code review."*

I focus on software because I think it is commonly trusted far out of proportion with its level of validation.  Everyone understands that physical measurements must be validated, devices must be calibrated, experiments should ideally be reproduced in other labs, etc.-- but code seems to be a cultural blind spot in this regard.

When a computational result can be directly compared to an experimental result, then of course agreement should increase trust in both.  More commonly, I think, a given result arises from a combination of experiment ("data collection") and computation ("data analysis"), and comparisons can only be made between attempts incorporating both.  Again agreement from multiple attempts should increase trust--but only if the analysis steps lack common components.  This is another reason to focus on software: software is typically used downstream of data collection, so a bug can easily mask whatever signal is present in the underlying data, producing spurious agreement or spurious disagreement in the final result.  Because software often has the last word in generating a result, then, it demands an even higher level of trust than upstream inputs.

Code review is certainly a good thing, but in my view is never sufficient to generate trust.  Anecdotally, I've found plenty of bugs in code that was already reviewed.  In any case, "code review" means many things to many people, and obviously the likelihood of finding bugs varies widely with the skill of the reviewer.

*"Second, this statement is not only true for software, it is also true for this complete paper. In order to believe the chances for errors claimed here, this paper itself needs to be verified, and not at the level of each assumption made internally (in the "How frequently ..." section), but at the level of the overall claim. This is not easy, but it would be worthwhile, similar to the author's statement, "Measuring such values rigorously in different contexts would be valuable but also tremendously difficult" (but at a different level). If "most scientific results are probably wrong," the author should be able to select a relatively small number of papers and demonstrate how software errors led to wrong results. I would like to see such an experiment, which would serve to verify this paper, rather than it standing as an unverified claim about verification."*

This is an opinion piece; I hope the more speculative language now makes clear that I am expressing justifiable anxiety that a serious problem may exist, rather than asserting that it definitely does exist.  I certainly agree that verifying my estimates would be a great thing to do (particularly the aggregate error rate, not just the individual factors, as you point out).  However I think that would be a major undertaking that is not tractable for me to do in this paper.

I do already cite a number of cases where software bugs resulted in wrong results, but these are basically anecdotal, and of course they are the ones that have already been found and reported.  The proportion of these to the overall literature is vanishingly small.  There are surely many more papers where an author or reader is privately aware of an error.  And a still much larger proportion of papers, I believe (but cannot prove), contain errors that remain completely unknown.

I can think of only two ways to determine that proportion empirically.  The first is to identify existing attempts to reproduce results, confirm that they are not subject to common sources of error, and track down the causes of any disagreement.  This method may be subject to selection bias (i.e. in general, only important or controversial results get replication attempts in the first place).

The second is to take a random sample of papers and attempt to fully reproduce them, or at least to carefully review the code in search of errors.  That would be really a lot of work-- in one example, an independent reproduction of a single computational study took 3 months.  A systematic campaign to reproduce computational results would be great, inspired by similar efforts focusing on reproducing experimental results (e.g. the Amgen study and the OSF Reproducibility Project).

But I can't take it on alone!  Rather I hope this paper helps to demonstrate the need for researchers, funders, and publishers to take code verification more seriously, and to foster the reproduction studies that would be needed to confirm or deny my estimates.  Crucially, it's not just a matter of successfully running a study author's code (which, in the example case of in ACM conferences and journals, can be downloaded and compiled for only about half of the papers anyway). Journal policies requiring at least that level of replication would be a good start.  But really the point here is to use different code to generate the same result.

So I think we agree: I am making an unverified claim about verification, and I too would like to see it verified.

*"Finally, there is the classic problem with verification of a model (software, in this case): that fact that it works well in one domain is no guarantee that it will work well in another domain."*

True, we can never make absolute guarantees.  But we can do better than the status quo, which all too often provides no verification at all.  Also, this point opens the question of the breadth of applicability of a given software artifact: some software does only a very specific thing, and so can be thoroughly verified within its single domain, while other software is very generic and so is much harder to verify across domains.  I don't address this in the paper, except to the extent that the factor for "proportion of lines executed" is tangentially related (e.g., successful tests exercising some code paths say nothing about runs taking different code paths).  That factor could be thought of more abstractly as the likelihood that verification in one domain should generate trust in another.

*"Having made these objections to the degree of the illness of the patient, I mostly agree with remedies discussed in the last section.  Open available of data and code is clearly good for both trust and reproducibility.  Running (computational) experiments multiple ways can help finds any errors in any one of them, assuming they do not use common components (e.g., libraries, tools, or even hardware) that could lead to systematic biases.  But how this should be done is less clear.  For example, we have enough workflow systems that I don’t see any need for any one of them to be more trusted than the code that runs on them; we can just use different workflow systems with different code as part of the testing."*

I agree that the ideal way to gain trust in a particular result is to observe agreement from two experiments in which *everything* differs, even the workflow system and the hardware (at some length: http://davidsoergel.com/posts/confirmation-depth-as-a-measure-of-reproducible-scientific-research, but just see Fig. 2 there for the main point).

However: if we could agree on a common, trusted workflow system, that would make it much easier both to verify software components and to track down sources of error, simply by swapping out individual components of workflows with purportedly equivalent alternative implementations.  When components are reused across workflows (and across labs, etc.), crowdsourced results from such component-swap experiments would quickly reveal which components are most commonly associated with robust results.  I'll have to describe that vision more thoroughly elsewhere, but for now I hope it points at one thing I hope we could gain from standardizing workflow and provenance descriptions.  Perhaps more simply: researchers are more likely to examine (and perhaps tweak and reuse) a workflow written in a language (or graphical notation, etc.) with which they are already familiar; Balkanization of workflow systems largely defeats their purpose.

*"Back to the author’s last point, I agree that "to recognize the urgent need" is essential, but to me, the need is verification; I could read this closing comment as saying that the need is widely adopted and widely trusted workflow tools.  This should be clarified."*

I did really mean both things--I've tried to clarify that.

Thanks again for the very helpful comments!

# Peer review: Referee response for version 1

**DOI:** 10.5256/f1000research.6338.r7019

**Published:** 2014-12-16

**Authors:** Titus Brown

**Affiliations:** 1 Microbiology and Molecular Genetics, Michigan State University, East Lansing, MI, USA

David Soergel's opinion piece applies numerical calculations and common (software engineering) sense to thinking about errors in scientific software.  I have seen no other piece that so simply and brutally summarizes the likely problems with current software development approaches in science, and I wholeheartedly agree with his recommendations.  I think that the recommendation for a common trusted workflow system is an interesting one; I am particularly impressed by the point that we need separate implementations of important software, as this is often neglected by funding agencies and non-computational scientists.

The large majority of the points in the paper are well taken and should not be controversial except perhaps in aggregate!

The only major flaw in the paper is an overstatement of the central thesis.  For example,

- The title is too definite; it needs a "probably" (which may decrease pithiness);
- Same with the abstract. One fix might be to eliminate the first sentence and move the last sentence to the top.
- For scenario 1, it's a pity there are no citations for these numbers, because they are nonintuitive (I found 20% executed to be too low, until I really thought it through, and then I agreed; but I'm not sure many people will believe). Is there any way to either bound or "suppose" these numbers a bit more?

I would say that if the statements can be softened a bit to indicate that all of this is *almost 100% certainly the case but we can't actually say it definitely* then the article would be very acceptable.

I didn't see the reference to the sign error debacle at the appropriate "sign error" point in the paper:

http://boscoh.com/protein/a-sign-a-flipped-structure-and-a-scientific-flameout-of-epic-proportions.html

Another reference that could be usefully added, given space: http://www.fastcompany.com/28121/they-write-right-stuff

It might be worth adding a reference to the recent SSH debacle, where it turned out that incredibly well used software had a significant flaw.  In other words, it's not enough for software to be well used for it to be correct!  (Space permitting.)

I have read this submission. I believe that I have an appropriate level of expertise to confirm that it is of an acceptable scientific standard, however I have significant reservations, as outlined above.

# Response

**Published:** 2014-12-18

**Authors:** David Soergel

Thanks for the kind and helpful comments!  The editors prefer to wait for more reviews before issuing a revision, but in the meantime:

1. In the title, I could go with "...may undermine...".  (The loss in pithiness is indeed a shame, but so be it).
2. Agreed re rearranging and toning down the abstract.
3. I'll do another search for references and justifications for the ballpark estimates of % LOC executed and so on, but I expect this one will be hard because there's so much variation.  For now the numbers are just my intuitions based on experience; I can try to clarify that at least.  I think the fuzziest one is the plausibility term-- I know of no effort to measure that, and am not even sure how you'd go about it.  In the course of code development and data analysis, how often do you look at a result and think "that's just not right"?  That one varies too with the paranoia level of the scientist.  (e.g., I'm a big fan of doing sanity checks that may reveal that some result is not plausible, even if that fact was not immediately obvious).

Thanks for the citation suggestions.  They're both already in there, actually (15 and 3, respectively), but I'll cite them from additional places as you suggest.

And yes, I'll add a para mentioning "Linus's Law" ("Given enough eyeballs, all bugs are shallow") and the recent counterexamples (Heartbleed, Goto Fail, and Shellshock).  These are notable because they're all security vulnerabilities, which (perhaps rightly) get a lot more press than bugs of other kinds.  There's also a world of difference between widespread *usage* and widespread *reading the code*-- a distinction that is sometimes glossed over in these discussions.

Thanks again for the comments!

# Response

**Published:** 2015-07-20

**Authors:** David Soergel

Thanks again for the comments, and apologies for the long-delayed response.  The changes described above are reflected in the new version.
